# Supplementary material for: Mechanistic basis of atypical TERT promoter mutations
Source: Nat Commun. 2024 Nov 18;15:9965. doi: 10.1038/s41467-024-54158-5 (PMC11574208; doi:10.1038/s41467-024-54158-5)
Supplement: Supplementary file 1 — Supplementary Information [file 41467_2024_54158_MOESM1_ESM.pdf]

Supplementary appendix

Table of Contents

Supplementary Figure 1 .... *2*
Supplementary Figure 2 .... *3*
Supplementary Figure 3 .... *4*
Supplementary Figure 4 .... *5*
Supplementary Figure 5 .... *6*
Supplementary Figure 6 .... *7*
Supplementary Table 1 .... *8*
Supplementary Table 2 .... *9*
Supplementary Table 3 .... *10*
Supplementary Table 4 .... *11*
Supplementary Table 5 .... *12*
Supplementary Table 6 .... *13*
Supplementary references *14*

Supplementary Figure 1

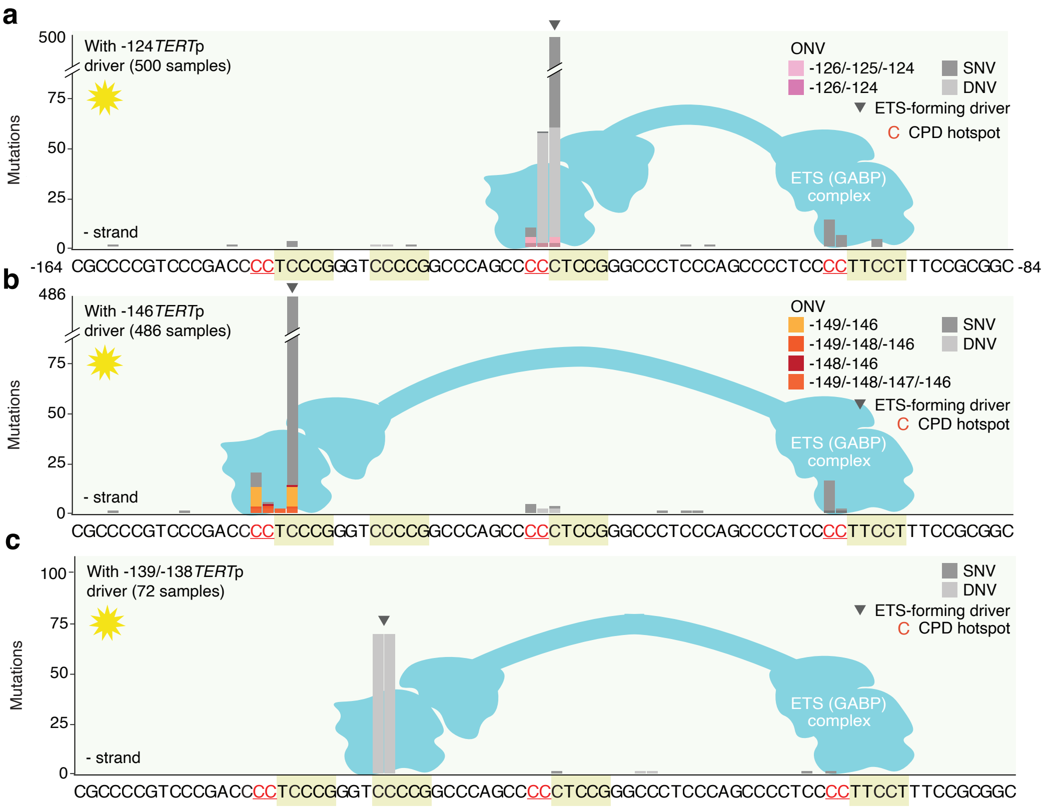

Supplementary Figure 1. **Atypical mutations co-occur with each of the three ETS-forming *TERT*p driver mutations**. GENIE samples in the "High *TERT*p, UV" subcohort were further subdivided based on the individual driver mutations: (**a**) -124 bp, (**b**) -146 bp and (**c**) -139/-138 bp. Mutations at the native ETS site co-occurred with all three driver mutations, although to a limited extent in the -139/-138 bp sample subset. Notably, four of the mutations at -126 bp occurred in patients having a -146 bp driver mutation. Source data are provided as a Source Data file.

Supplementary Figure 2

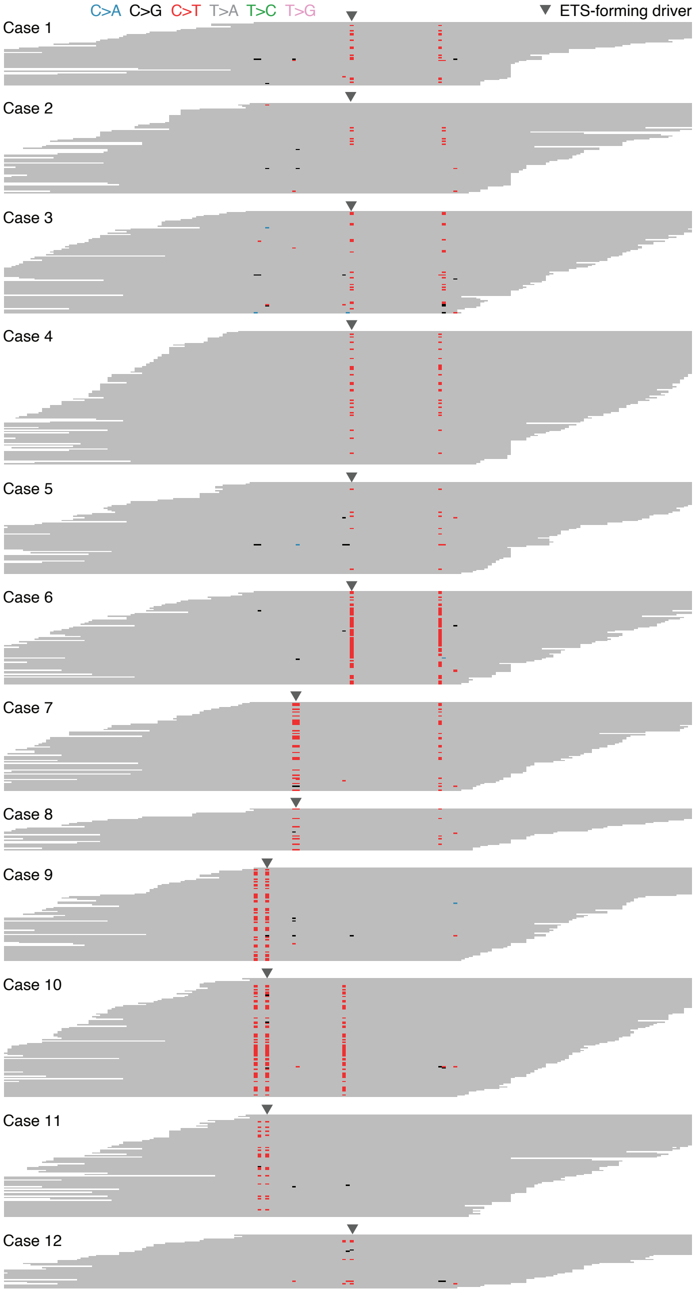

Supplementary Figure 2. Atypical mutations co-occur in cis with driver mutations in 100k Genomes melanoma whole-genome sequencing data. Sequencing reads from 100k Genones melanoma cases with atypical mutations at one or more positions of interest (-97, -100, -101, -126, -138, -148 or -149 bp from at ATG codon). ETS-forming driver mutations (-124, -146, -139/-138) are indicated with a triangle. Only reads which covered all bases between -97 and -149 bp were considered shown. Mutations only at the specific positions of interest are indicated, with substitutions coloured by pyrimidine-based mutation type. Source data are provided as a Source Data file.

Supplementary Figure 3

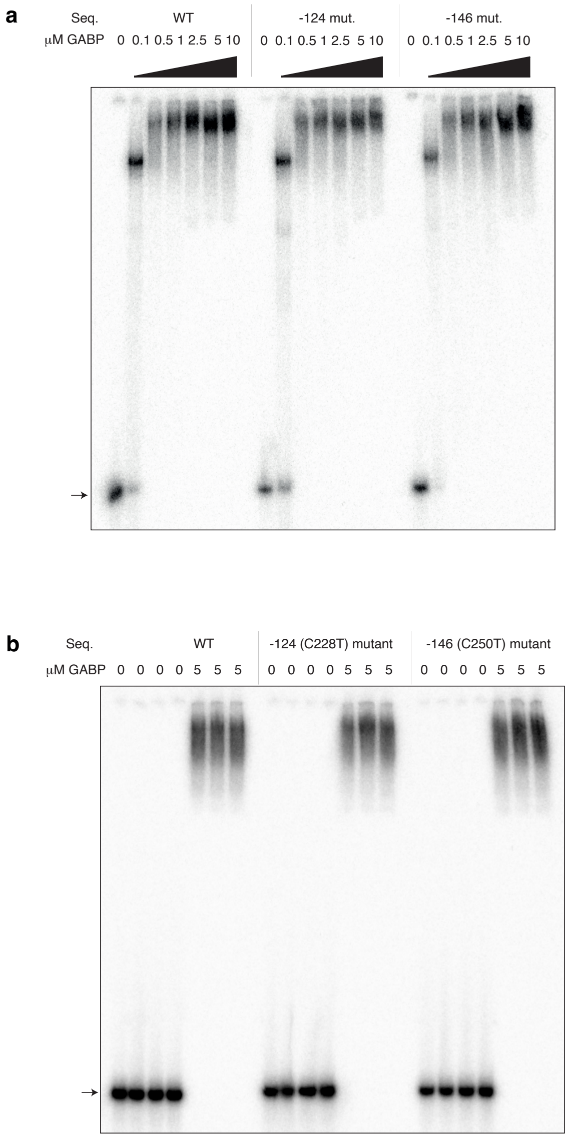

Supplementary Figure 3. Electrophoretic mobility shift assay (EMSA) showing GABP complex binding to *TERT*p dsDNA oligonucleotides. (**a**) Concentration curve of GABPA/B heterodimers showing an incomplete shift at 0.1 mM and complete shift at 0.5 mM which improves with increasing concentration. 5 mM was chosen as the concentration for UV footprinting. (**b**) EMSA of samples used in UV footprinting experiment (main **Fig. 5b**). Arrowheads indicate unbound oligo. Gels are representative of experiments performed at least in triplicate. Exact genomic coordinates of the oligos are chr5:1295183-1295272 (hg19) and chr5:1295068-1295157 (hg38). Source data are provided as a Source Data file.

Supplementary Figure 4

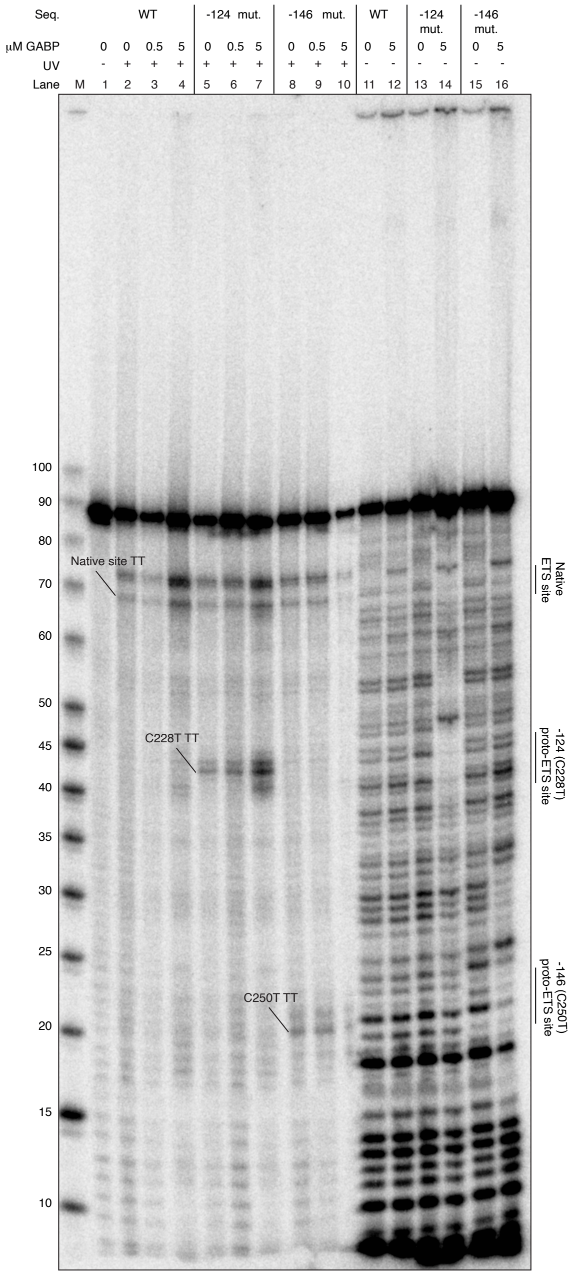

Supplementary Figure 4. DNase I footprinting of the *TERT*p. T4 endo V digestion is shown side by side, revealing the location of CPD-prone TT dinucleotides at the native ETS and ETS proto-sites, thus allowing these sites to be mapped to the footprint. UV footprint samples (independent replicates) are shown in lanes 1-10 and DNAse experiments with or without 5 μM of GABPA/B heterodimers in 11-16. Note that the lane 10 sample was partially lost in phenol chloroform extraction. The gel is representative of two repeated experiments. M is the marker lane showing a 10-100 bp ladder (Affymetrix). The labelled oligos are the same as in **Supplementary Fig. 3**. Source data are provided as a Source Data file.

Supplementary Figure 5

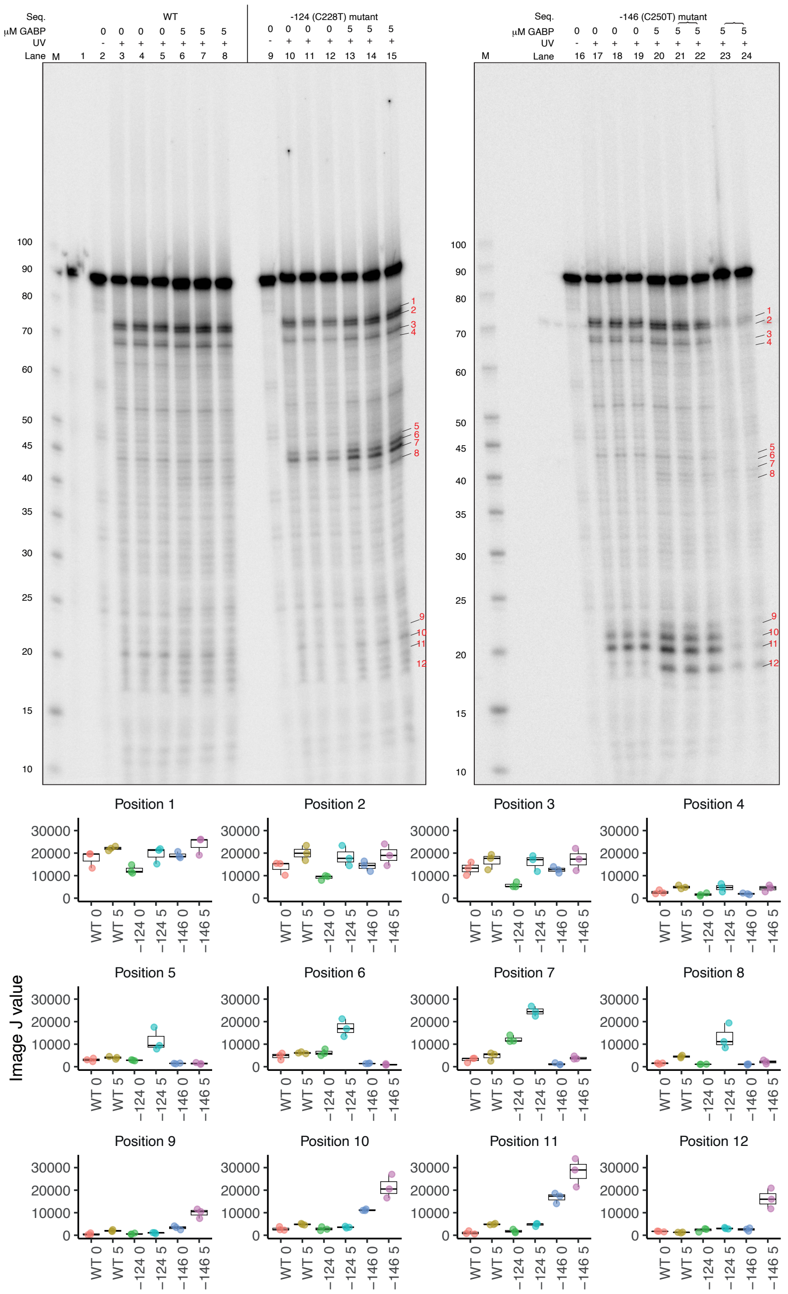

Supplementary Figure 5. CPD formation levels following UV irradiation of *TERT*p dsDNA fragments. Complete gel image to complement main **Fig. 5**. Note that lanes 21/22 and 23/24 were repeated samples. Sample 3 for the -146 bp mutant oligo (lanes 23/24) was partially lost during phenol chloroform extraction but shows a clear UV hotspot. For main **Fig. 5a** only the first two replicates (Lanes 20 and 21) are shown. Gel is representative of more than three replicate experiments. M is marker lane showing 10-100 bp ladder (Affymetrix). Bands indicated by the numbers on the red numbers on the gel were quantified using ImageJ Analyse tool. Boxplots represent median and first and third quartiles (25th and 75th percentiles), with whiskers indicating 1.5 times the interquartile range for the three replicates. Individual values are shown as dots and coloured by sample type (WT, -124 or -146 *TERT*p sequence with or without 5 uM GABP proteins). The labelled oligos are the same as in **Supplementary Fig. 3**. Source data are provided as a Source Data file.

Supplementary Figure 6

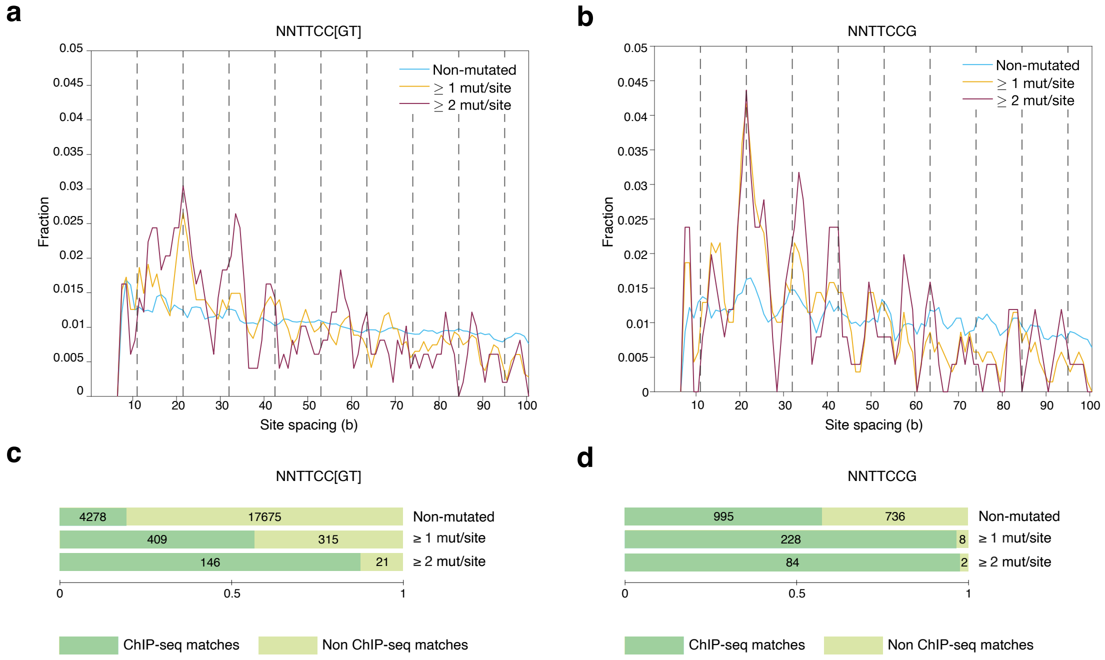

Supplementary Figure 6. Helical periodicity in the spacing of somatically mutated GABP binding site pairs. (**a**) Smoothened histogram showing the distribution of distances for co-localized (within 100 bp) ETS motif match pairs (NNTTCCK) in 1000 bp upstream regions genome wide. (**b**) Same as **a** but for a subset of site pairs matching the motif NNTTCCG. Distributions were computed for non-mutated pairs (none of the sites mutated), pairs where both sites had at least one mutation (>= 1 mut/site) as well as pairs where both sites had more than two mutations (>= 2 mut/site). Pairs where the two sites overlapped were excluded from the analysis. The dashed lines indicate a periodicity of 10.5 bp. (**c**) The proportion of pairs where both sites were positioned within a strong GABP ChIP-seq peak, for the motif NNTTCCK, shown for non-mutated pairs as well as pairs with at least one or two mutations per site. (**d**) Same as **c** but for the motif NNTTCCG. Source data are provided as a Source Data file.

Supplementary Table 1

From ATG (bp)	Variant	hg19 chr5 coord	hg38 chr5 coord	Comment	Creates ETS?	Reported in
-46	C>T	1295150	1295035		No	1
-57	T>G	1295161	1295046	Recurrent in bladder cancer	Yes	2
-58	C>T	1295162	1295047		No	1
-87	C>T	1295191	1295076		No	3
**-97**	**C>T**	**1295201**	**1295086**	**Recurrent in skin cancer**	**No**	**4,5**
**-100**	**C>T**	**1295204**	**1295089**	**Recurrent in skin cancer**	**No**	**1,4,5**
**-101**	**C>T**	**1295205**	**1295090**	**Recurrent in skin cancer**	**No**	**1,4,5**
-113	C>T	1295217	1295102		No	4
-124	C>T	1295228	1295113	Driver	Yes	1,2,4-13
**-125/-124**	**CC>TT**	**1295229**	1295114	**Recurrent in skin cancer**	**No**	**1,4,7,11,12**
**-126**	**C>T**	**1295230**	1295115	**Recurrent in skin cancer**	**No**	**1,4,10-12**
**-139/-138**	**CC>TT**	**1295243/42**	1295128	**Likely driver in skin cancer**	**Yes**	**1,2,4,6,7,11-13**
-144	C>T	1295248	1295133		No	1
-146	C>T	1295250	1295135	Driver	Yes	1,2,4-13
**-149**	**C>T**	**1295253**	1295138	**Recurrent in skin cancer**	**No**	**1,4-7,11**
-154	C>T	1295258	1295143		No	1
-156	C>T	1295260	1295145		Yes	1
-176	C>T	1295280	1295165		No	1
-187	C>T	1295291	1295176		No	1

Supplementary Table 1. Survey of previously reported somatic mutations in the TERT promoter. Known drivers are underlined, recurrent mutations found only in skin cancers are shown in bold, and less recurrent mutations are shown in grey. Positions are labelled with the number of bases from the ATG codon (hg19 chr5:1295104 and hg38 chr5:1294989), as well as their respective genome coordinates.

Supplementary Table 2

	Cancer subtypes	Samples	% of GENIE v11
**Total in GENIE v11**	756	118094	100
**Remaining after removal of:**			
Samples using assay lacking *TERT*p or with reduced targeted region size (<300,000 bp)	520	46341	39.2
Non-unique tumors (one sample per patient)	513*	41617	35.2
Subtypes with <40 samples	128	38267	32.4
Subtypes with <500 mutations	81	34832	29.5
Subtypes with <1% *TERT*p mutation	59	19755	16.7

Supplementary Table 2. Summary of included samples from GENIE v11. Cancer subtypes refer to OncoTree codes as registered in GENIE. The final set consisted of unique samples (one tumor per patient). To allow analysis of mutational signatures, mutation burden and *TERT*p mutations, filters were applied to exclude cancer subtypes that were small, had low mutations counts, or where capture assays lacking *TERT*p or with limited genomic coverage were used. *, Some cancer subtypes with low patient counts were removed at this stage.

Supplementary Table 3

Mutation	High *TERT*p, UV		High *TERT*p, no UV		Low *TERT*p	
	**Samples**	**+Driver**	**Samples**	**+Driver**	**Samples**	**+Driver**
*TERT*p driver mutations						
-124_SNV	438		2252		377	
-125/-124_DNV	57		1		2	
-146_SNV	473		528		81	
-139/-138_DNV	72		7		6	
*TERT*p driver with ONVs						
-126/-125/-124_ONV	2		0		0	
-126/-124_ONV	3		1		1	
-148/-146_ONV	1		0		0	
-149/-146_ONV	9		0		0	
-149/-148/-146_ONV	1		0		0	
-149/-148/-147/-146_ONV	2		0		0	
*TERT*p proto-ETS atypical						
-125 SNV	1	1 (1,0,0)	0	0	1	0
-126_SNV	9	9 (5,4,0)	0	0	1	1 (0,1,0)
-149_SNV	8	8 (0,8,0)	0	0	1	0
-148_SNV	1	1 (0,1,0)	0	0	0	0
*TERT*p native ETS atypical						
-97_SNV	4	4 (4,0,0)	0	0	0	0
-100_SNV	8	7 (6,1,0)	0	0	1	0
-101/-100_DNV	2	1 (0,1,0)	0	0	0	0
-101_SNV	30	29 (14,14,1)	0	0	5	5 (2,3,0)
Summary						
All drivers	1058		2789		467	
Proto-ETS atypical ONV	18	18 [100]	1	1 [100]	1	1 [100]
Proto-ETS atypical SNV	19	19 [100]	0	0 [0]	3	1 [33]
All proto-ETS atypical	37	37 [100]	1	1 [100]	4	1 [25]
All native ETS atypical	44	41 [93.2]	0	0 [0]	6	5 [83]
**Total samples**	1569		4065		14121	

Supplementary Table 3. Overview of driver and atypical mutations in GENIE subcohorts. Total sample counts for each mutation type in "High *TERT*p, UV" (≥20% of samples having *TERT*p driver mutations, ≥10% of mutations in subcohort attributed to SBS7 or DBS1), "High *TERT*p, No UV" (≥20% *TERT*p, <10% UV mutations) and "Low *TERT*p" (<20% *TERT*p) GENIE subcohorts. Mutations are either single nucleotide variants (SNV), double nucleotide variants (DNV) or oligonucleotide variants (ONV). Atypical mutations were split into proto-ETS site and native site mutations. The "+Driver" columns indicate the subset of atypical mutations that co-exist with *TERT*p driver events in the same samples, where numbers in parentheses further specify co-occurence with -124, -146 and DNV -139/-138 driver mutations, respectively. In "Summary", the percent atypical mutations co-existing with a driver mutation is in square brackets.

Supplementary Table 4

Cancer type	Samples	-139/-138 CC>TT	Frequency (%)	Mutational signature	Etiology
Cutaneous Melanoma	870	48	5.51	DBS1	UV
Melanoma of Unknown Primary	175	9	5.14	DBS1	UV
Melanoma	124	6	4.84	DBS1	UV
Cutaneous Squamous Cell Carcinoma	115	4	3.48	DBS1	UV
Bladder Urothelial Carcinoma	1282	4	0.31	DBS11[14]	APOBEC
Basal Cell Carcinoma	43	3	6.98	DBS1	UV
Cancer of Unknown Primary	525	3	0.57	?	?
Squamous Cell Carcinoma, NOS	80	2	2.50	DBS1	UV
Upper Tract Urothelial Carcinoma	258	2	0.78	DBS11[14]	APOBEC

Supplementary Table 4. Cancer subtypes with recurrent (*n* > 1) -139/-138 CC>TT mutations had active double base signatures resulting from UV light (DBS1) and APOBEC (DBS11) mutagenesis. Mutational signatures identified from Alexandrov et al [14]. DBS1 relates to CC>TT mutations which result from UV light exposure. DBS11 is characterized by mainly CC>TT mutations with the likely etiology of APOBEC mutagenesis.

Supplementary Table 5

Oligos
*TERT*p WT F 90 (C-rich)<br>ACCCCGCCCCGTCCCGACCCCTCCCGGGTCCCCGGCCCAGCCCCCTCCGGGCCCTCCCAGCCCCTCCCCTTCCTTTCCGCGGCCCCGCCC
*TERT*p WT R 90 (G-rich)<br>GGGCGGGGCCGCGGAAAGGAAGGGGAGGGGCTGGGAGGGCCCGGAGGGGGCTGGGCCGGGGACCCGGGAGGGGTCGGGACGGGGCGGGGT
*TERT*p 124 F 90 (C-rich)<br>ACCCCGCCCCGTCCCGACCCCTCCCGGGTCCCCGGCCCAGCCCCTTCCGGGCCCTCCCAGCCCCTCCCCTTCCTTTCCGCGGCCCCGCCC
*TERT*p 124 R 90 (G-rich)<br>GGGCGGGGCCGCGGAAAGGAAGGGGAGGGGCTGGGAGGGCCCGGAAGGGGCTGGGCCGGGGACCCGGGAGGGGTCGGGACGGGGCGGGGT
*TERT*p 146 F 90 (C-rich)<br>ACCCCGCCCCGTCCCGACCCCTTCCGGGTCCCCGGCCCAGCCCCCTCCGGGCCCTCCCAGCCCCTCCCCTTCCTTTCCGCGGCCCCGCCC
*TERT*p 146 R 90 (G-rich)<br>GGGCGGGGCCGCGGAAAGGAAGGGGAGGGGCTGGGAGGGCCCGGAGGGGGCTGGGCCGGGGACCCGGAAGGGGTCGGGACGGGGCGGGGT

Supplementary Table 5. Oligonucleotides for CPD/DNase footprinting and gel-shift assays. Exact genomic coordinates of the fragment (hg19 chr5:1295183-1295272, hg38 chr5:1295068-1295157).

Supplementary Table 6

Gene	Position hg19	Forward 5´- 3´	Reverse 5´- 3´	Amplicon
TERT	chr5:1295152-1295311	AGCGCTGCCTGAAACTCG	CCTGCCCCTTCACCTTCCAG	160 bp

Primer	Sequence 5´- 3´
TERT Fwd with barcode	GGACACTCTTTCCCTACACGACGCTCTTCCGATCT**NNNNNNNNNNNN**ATGGGAAAGA GTGTCC*AGCGCTGCCTGAAACTCG*
TERT Rev	GTGACTGGAGTTCAGACGTGTGCTCTTCCGATCT*CCTGCCCCTTCACCTTCCAG*
Illumina Fwd	AATGATACGGCGACCACCGAGATCTACACTCTTTCCCTACACGACGCTCTTCCGATCT
Illumina Rev with index	CAAGCAGAAGACGGCATACGAGATNNNNNNGTGACTGGAGTTCAGACGTGTGCTCT TCCGATCT

Supplementary Table 6. Primer sequences used for SiMSen-seq in *TERT*p. Target specific *TERT*p primer is shown in the top panel and indicated in italics in in the SiMSen primer below. 12 bp barcode (NNNNNNNNNNNN) in the *TERT* Fwd primer is shown in bold. Index sequence in the Illumina Rev primer is shown with underline. Hg38 position chr5:1295037-1295196.

Supplementary references

1. Heidenreich, B. *et al.* Telomerase reverse transcriptase promoter mutations in primary cutaneous melanoma. *Nat Commun* **5**, 3401 (2014). https://doi.org/10.1038/ncomms4401
2. Hurst, C. D., Platt, F. M. & Knowles, M. A. Comprehensive mutation analysis of the TERT promoter in bladder cancer and detection of mutations in voided urine. *Eur Urol* **65**, 367-369 (2014). https://doi.org/10.1016/j.eururo.2013.08.057
3. Huang, D. S. *et al.* Recurrent TERT promoter mutations identified in a large-scale study of multiple tumour types are associated with increased TERT expression and telomerase activation. *Eur J Cancer* **51**, 969-976 (2015). https://doi.org/10.1016/j.ejca.2015.03.010
4. Zehir, A. *et al.* Mutational landscape of metastatic cancer revealed from prospective clinical sequencing of 10,000 patients. *Nat Med* **23**, 703-713 (2017). https://doi.org/10.1038/nm.4333
5. Gupta, S. *et al.* A Pan-Cancer Study of Somatic TERT Promoter Mutations and Amplification in 30,773 Tumors Profiled by Clinical Genomic Sequencing. *J Mol Diagn* **23**, 253-263 (2021). https://doi.org/10.1016/j.jmoldx.2020.11.003
6. Fredriksson, N. J., Ny, L., Nilsson, J. A. & Larsson, E. Systematic analysis of noncoding somatic mutations and gene expression alterations across 14 tumor types. *Nat Genet* **46**, 1258-1263 (2014). https://doi.org/10.1038/ng.3141
7. Horn, S. *et al.* TERT promoter mutations in familial and sporadic melanoma. *Science* **339**, 959-961 (2013). https://doi.org/10.1126/science.1230062
8. Huang, F. W. *et al.* Highly recurrent TERT promoter mutations in human melanoma. *Science* **339**, 957-959 (2013). https://doi.org/10.1126/science.1229259
9. Killela, P. J. *et al.* TERT promoter mutations occur frequently in gliomas and a subset of tumors derived from cells with low rates of self-renewal. *Proc Natl Acad Sci U S A* **110**, 6021-6026 (2013). https://doi.org/10.1073/pnas.1303607110
10. Shain, A. H. *et al.* Genomic and Transcriptomic Analysis Reveals Incremental Disruption of Key Signaling Pathways during Melanoma Evolution. *Cancer Cell* **34**, 45-55 e44 (2018). https://doi.org/10.1016/j.ccell.2018.06.005
11. Vinagre, J. *et al.* Frequency of TERT promoter mutations in human cancers. *Nat Commun* **4**, 2185 (2013). https://doi.org/10.1038/ncomms3185
12. Andres-Lencina, J. J. *et al.* TERT promoter mutation subtypes and survival in stage I and II melanoma patients. *Int J Cancer* **144**, 1027-1036 (2019). https://doi.org/10.1002/ijc.31780
13. Allory, Y. *et al.* Telomerase reverse transcriptase promoter mutations in bladder cancer: high frequency across stages, detection in urine, and lack of association with outcome. *Eur Urol* **65**, 360-366 (2014). https://doi.org/10.1016/j.eururo.2013.08.052
14. Alexandrov, L. B. *et al.* The repertoire of mutational signatures in human cancer. *Nature* **578**, 94-101 (2020). https://doi.org/10.1038/s41586-020-1943-3
