# Supplementary material for: Mechanistic basis of atypical TERT promoter mutations
Source: Nat Commun. 2024 Nov 18;15:9965. doi: 10.1038/s41467-024-54158-5 (PMC11574208; doi:10.1038/s41467-024-54158-5)
Supplement: Supplementary file 2 — Description of Additional Supplementary Files [file 41467_2024_54158_MOESM2_ESM.pdf]

Description of Additional Supplementary Files

File Name: Supplementary Data 1
Description: Code/scripts underlying analyses in this study
