# Supplementary material for: Mechanistic basis of atypical TERT promoter mutations
Source: Nat Commun. 2024 Nov 18;15:9965. doi: 10.1038/s41467-024-54158-5 (PMC11574208; doi:10.1038/s41467-024-54158-5)
Supplement: Supplementary file 3 — Supplementary Data 1 [file 41467_2024_54158_MOESM3_ESM.zip › Code/ICGC/data/ChIP/readme.rtf]

A549: SL6015 & SL6595GM12878: SL205 & SL203HeLa-S3: SL610 & SL611HepG2: SL275 & SL276HL60: SL16321 & SL12633K562: SL3356 & SL2943MCF-7: SL14683 & SL13459SK-N-SH: SL13752 & SL12627
