# Supplementary material for: Mechanistic basis of atypical TERT promoter mutations
Source: Nat Commun. 2024 Nov 18;15:9965. doi: 10.1038/s41467-024-54158-5 (PMC11574208; doi:10.1038/s41467-024-54158-5)
Supplement: Supplementary file 4 — Reporting Summary [file 41467_2024_54158_MOESM4_ESM.pdf]

nature portfolio

Corresponding author(s): Larsson

Last updated by author(s): Oct 10, 2024

Reporting Summary

Nature Portfolio wishes to improve the reproducibility of the work that we publish. This form provides structure for consistency and transparency in reporting. For further information on Nature Portfolio policies, see our Editorial Policies and the Editorial Policy Checklist.

Statistics

For all statistical analyses, confirm that the following items are present in the figure legend, table legend, main text, or Methods section.

n/a	Confirmed	
☐	☒	The exact sample size (*n*) for each experimental group/condition, given as a discrete number and unit of measurement
☐	☒	A statement on whether measurements were taken from distinct samples or whether the same sample was measured repeatedly
☐	☒	The statistical test(s) used AND whether they are one- or two-sided<br>*Only common tests should be described solely by name; describe more complex techniques in the Methods section.*
☒	☐	A description of all covariates tested
☒	☐	A description of any assumptions or corrections, such as tests of normality and adjustment for multiple comparisons
☐	☒	A full description of the statistical parameters including central tendency (e.g. means) or other basic estimates (e.g. regression coefficient) AND variation (e.g. standard deviation) or associated estimates of uncertainty (e.g. confidence intervals)
☐	☒	For null hypothesis testing, the test statistic (e.g. *F*, *t*, *r*) with confidence intervals, effect sizes, degrees of freedom and *P* value noted<br>*Give P values as exact values whenever suitable.*
☒	☐	For Bayesian analysis, information on the choice of priors and Markov chain Monte Carlo settings
☒	☐	For hierarchical and complex designs, identification of the appropriate level for tests and full reporting of outcomes
☐	☒	Estimates of effect sizes (e.g. Cohen's *d*, Pearson's *r*), indicating how they were calculated

Our web collection on statistics for biologists contains articles on many of the points above.

Software and code

Policy information about availability of computer code

Data collection	No code/software was used for data collection.
Data analysis	Simsen data was analysed with UMIErrorCorrect v0.28 (https://github.com/stahlberggroup/umierrorcorrect/). GENIE v11 data was downloaded from Synapse and analysed with R (version 4.1.0), using deconstructSigs (1.9.0), Genomics England data was analysed inside the research environment using Python version 3.10.4, Bash version 4.4.20 and R. Once through the airlock Genomics England data, as well as the exported Simsen data was analysed and visualised using MATLAB (release r2023b). ICGC and ENCODE data was analysed with MATLAB (release r2023b). Scripts are available as a supplementary zip archive.<br>Gels were analysed with Multi Gauge (version 3.0) and Image J (Fiji version 2.1.0/1.53c).

For manuscripts utilizing custom algorithms or software that are central to the research but not yet described in published literature, software must be made available to editors and reviewers. We strongly encourage code deposition in a community repository (e.g. GitHub). See the Nature Portfolio guidelines for submitting code & software for further information.

Data

Policy information about availability of data

All manuscripts must include a data availability statement. This statement should provide the following information, where applicable:

- Accession codes, unique identifiers, or web links for publicly available datasets
- A description of any restrictions on data availability
- For clinical datasets or third party data, please ensure that the statement adheres to our policy

Sequencing data generated for this study has been deposited in SRA under BioProject PRJNA1062776 [https://www.ncbi.nlm.nih.gov/bioproject/PRJNA1062776].
Melanoma mutation data from the Australian Melanoma Genome Project via the ICGC data portal [https://docs.icgc-argo.org/docs/data-access/icgc-25k-data].
NCBI RefSeqAll gene annotations for hg19, via the UCSC table browser
[https://genome.ucsc.edu/cgi-bin/hgTables].
ENCODE ChIP-seq data for 8 cell lines (A549, GM12878, HeLa-S3, HepG2, HL-60, K562, MCF-7 and SK-N-SH) from UCSC
[http://hgdownload.cse.ucsc.edu/goldenPath/hg19/encodeDCC/wgEncodeHaibTfbs/].
GENIE v11 [https://www.synapse.org/#!Synapse:syn26706564].
Genomics England [https://www.genomicsengland.co.uk/research/research-environment].
Source Data are provided with this paper.

Research involving human participants, their data, or biological material

Policy information about studies with human participants or human data. See also policy information about sex, gender (identity/presentation), and sexual orientation and race, ethnicity and racism.

Reporting on sex and gender	N/A
Reporting on race, ethnicity, or other socially relevant groupings	N/A
Population characteristics	N/A
Recruitment	N/A
Ethics oversight	N/A

Note that full information on the approval of the study protocol must also be provided in the manuscript.

Field-specific reporting

Please select the one below that is the best fit for your research. If you are not sure, read the appropriate sections before making your selection.

☒ Life sciences ☐ Behavioural & social sciences ☐ Ecological, evolutionary & environmental sciences

For a reference copy of the document with all sections, see nature.com/documents/nr-reporting-summary-flat.pdf

Life sciences study design

All studies must disclose on these points even when the disclosure is negative.

Sample size	Available sequencing cohorts were analyzed. No statistics methods were used to determine sample size, as we sought to include as many samples as possible.
Data exclusions	Duplicate samples in GENIE stemming from the same patient were excluded, to ensure that mutation counts were unique/independent.
Replication	Gel analyses were repeated at least two times. Initial results from GENIE were replicated in 100k Genomes data.
Randomization	Randomization is not relevant for the computational and in vitro analyses performed in this study.
Blinding	No blinding was done. It is not possible to perform these experiments in a blinded fashion.

Reporting for specific materials, systems and methods

We require information from authors about some types of materials, experimental systems and methods used in many studies. Here, indicate whether each material, system or method listed is relevant to your study. If you are not sure if a list item applies to your research, read the appropriate section before selecting a response.

Materials & experimental systems

n/a	Involved in the study
☒	☐ Antibodies
☐	☒ Eukaryotic cell lines
☒	☐ Palaeontology and archaeology
☒	☐ Animals and other organisms
☒	☐ Clinical data
☒	☐ Dual use research of concern
☒	☐ Plants

Methods

n/a	Involved in the study
☒	☐ ChIP-seq
☒	☐ Flow cytometry
☒	☐ MRI-based neuroimaging

Eukaryotic cell lines

Policy information about cell lines and Sex and Gender in Research

Cell line source(s)	A375 melanoma cells, which were derived from a 54-year old female, were a gift from the Jonas Nilsson laboratory (University of Gothenburg).
Authentication	The cells were confirmed to carry the TERT C250T mutation as expected. No other authentication was performed.
Mycoplasma contamination	Not tested.
Commonly misidentified lines (See ICLAC register)	None to our knowledge.

Plants

Seed stocks	*Report on the source of all seed stocks or other plant material used. If applicable, state the seed stock centre and catalogue number. If plant specimens were collected from the field, describe the collection location, date and sampling procedures.*
Novel plant genotypes	*Describe the methods by which all novel plant genotypes were produced. This includes those generated by transgenic approaches, gene editing, chemical/radiation-based mutagenesis and hybridization. For transgenic lines, describe the transformation method, the number of independent lines analyzed and the generation upon which experiments were performed. For gene-edited lines, describe the editor used, the endogenous sequence targeted for editing, the targeting guide RNA sequence (if applicable) and how the editor was applied.*
Authentication	*Describe any authentication procedures for each seed stock used or novel genotype generated. Describe any experiments used to assess the effect of a mutation and, where applicable, how potential secondary effects (e.g. second site T-DNA insertions, mosiacism, off-target gene editing) were examined.*
